# Psychological Nursing of Patients With Stroke in China: A Systematic Review and Meta-Analysis

**DOI:** 10.3389/fpsyt.2020.569426

**Published:** 2020-12-11

**Authors:** Bingye Liao, Minni Liang, Qiuyi Ouyang, Hongqin Song, Xiaojun Chen, Yuejiao Su

**Affiliations:** Department of Operating Room, The First Affiliated Hospital of Sun Yat-sen University, Guangzhou, China

**Keywords:** psychological nursing, usual care, stroke, depressive symptoms, meta-analysis

## Abstract

The present study aimed to evaluate the efficacy of psychological nursing of patients with stroke in China. The Embase, PubMed, Cochrane Library, CNKI, and Wanfang databases were searched from inception to February 1, 2020. Randomized controlled trials (RCTs) reporting the efficacy of psychological nursing of patients with stroke were included. Revman 5.3 and Stata 15.0 were used for data analysis. Twelve RCTs and 1,013 patients with stroke were included in this systematic review and meta-analysis. The results revealed a significant difference in the Hamilton depression score between the psychological nursing and usual care groups. The meta-analysis of three studies (*n* = 235) that used a depressive symptom control of ≥25% as the outcome measure showed a significant difference between the two groups. In addition, significant differences were detected in the National Institute of Health stroke scale score and activities of daily living score between the two groups. The present meta-analysis suggests that in China, compared to the usual care, psychological nursing is more effective for alleviating depressive symptoms, improving neurological rehabilitation, and recovering the ability of daily life.

## Introduction

Social and psychological issues such as anxiety, depression, and cognitive impairment occur commonly after stroke in about 40% of the patients (1–3). Moreover, self-reported psychological conditions are prevalent, with ~60% of the patients with stroke feeling depressed and 67% feeling anxious (4). Psychological issues such as depression have a major impact on the quality of daily life (5) and are likely to occur over the first 26 weeks after stroke and predict poor functional outcomes at 52 weeks (6).

Nurses also face difficulty in coping with the fluctuating mood and uncooperative behavior of patients with stroke (7–9). Over half of the nurses and nearly half of the patients with stroke experience difficulties in interpersonal relationships (1). Thus, effective psychological care is necessary to improve nursing and to reduce the consumption and cost of healthcare (10).

Psychological nursing is the act of health professionals providing positive psychological interventions for patients after a medical event to help them acquire and maintain a good psychological state to ease rehabilitation and healthcare (11). Psychological nursing is of special importance for patients at high risk of illness-induced anxiety and depression (12). Psychological nursing is part of the shift of nursing toward the biological-psychological-social model (13). Psychological nursing does not only focus on the patient's illness but involves the entire nursing process (13). Its aim is to improve not only the patient's body comfort but also its psychological state, which is an important component of quality of life. Psychological nursing also affects the cognition of the patients through the behaviors and attitudes of the medical staff, improving their quality of life (14). It has been reported that psychological nursing can reduce perceived pain, anxiety, and depression in 82% of patients with advanced gastric cancer (15). The main difference between psychological interventions, such as cognitive-behavioral therapy and psychological nursing, is that cognitive-behavioral therapy is a definite treatment in time that requires a psychiatrist or psychologist while psychological nursing is an ongoing process (24 h/day, for the entire patient's hospitalization) that requires the active and continuous participation of all the nursing staff (11, 12). For now, psychological nursing is still in its infancy all over the world. Besides cultural and healthcare administrative differences among countries, the optimal psychological nursing interventions are still being pursued.

Presently, psychological nursing of patients with stroke is gaining increasing attention in China. Some studies compared psychological nursing to the usual care for patients with stroke (16–27), albeit the results were conflicting and no comprehensive analysis of these data are yet available. Therefore, systematic review and meta-analysis are essential to evaluate the efficacy of psychological nursing of patients with stroke in China.

## Methods

### Search Strategy

This systematic review was conducted according to the Preferred Reporting Items for Systematic Reviews and Meta-analyses (PRISMA) criteria (28). The Embase, PubMed, Cochrane Library, CNKI, and Wanfang databases were searched using the key words [“Psychological nursing” OR “Psychological care”] AND “Stroke” AND [“China” OR “Chinese”]. The databases were searched from their inception to February 1, 2020. We also screened the bibliographies of the retrieved studies and recent review articles to identify additional eligible trials. No language restrictions were applied.

### Inclusion and Exclusion Criteria

The following selection criteria were applied according to the PICOS principles. Participants (P): patients with stroke in China; Intervention (I): psychological nursing; Comparison (C): usual care; Outcomes (O): efficacy, which was evaluated using the Hamilton depression (HAMD) score, the National Institute of Health stroke scale (NINSS) score, and activities of daily living (ADL) score; Study design (S): random controlled trials (RCTs). Studies were excluded if they were published as reviews, editorials, letters, case reports, cell and animal studies, or expert opinions. Bingye Liao and Minni Liang independently and systematically screened the literature by titles and abstracts, and then read the full texts to determine the eligibility of the articles. If more than one article was from the same dataset, only the one with the largest sample size was included. Any disagreement in the literature search was resolved by the third author (Yuejiao Su).

### Data Extraction and Quality Assessment

All data from the selected studies were extracted independently by two reviewers (Qiuyi Ouyang and Xiaojun Chen), and any disagreement was resolved by the third reviewer (Hongqin Song). The extracted data, including the study year, number of samples, male, mean age (years) of the participants, and follow-up duration were summarized into simple standard forms. Each study was independently screened by two reviewers. The risk of bias assessment was evaluated according to the Cochrane Handbook for Systematic Reviews (29), which includes selective outcome, allocation concealment, blinding, incomplete outcome data, random generation, and other biases. Also, the quality of the included studies was assessed.

### Data Synthesis and Statistical Analysis

The continuous variable data were expressed as mean differences (MD) with 95% confidence intervals (CI). Odds ratio (ORs) and the 95% CI were calculated to combine the categorical variable data. Next, the missing standard deviations (SD) such as *P*-values or 95% CI were estimated if needed (29, 30). Among the combined study results, Cochran's Q test and the degree of inconsistency (*I*^2^) were used to assess heterogeneity (29). A fixed-effects model was used if *I*^2^ was <50%. Otherwise, the data were pooled using the random-effects method. All statistical analyses were performed in an intent-to-treat principle and using the RevMan 5.3 and Stata® Statistical Software Package, version 15.0 (StataCorp LP, College Station, TX, USA). The publication bias was estimated using the Egger's test. *P* < 0.05 indicated statistical significance.

## Results

### Search Results

**Figure 1:**
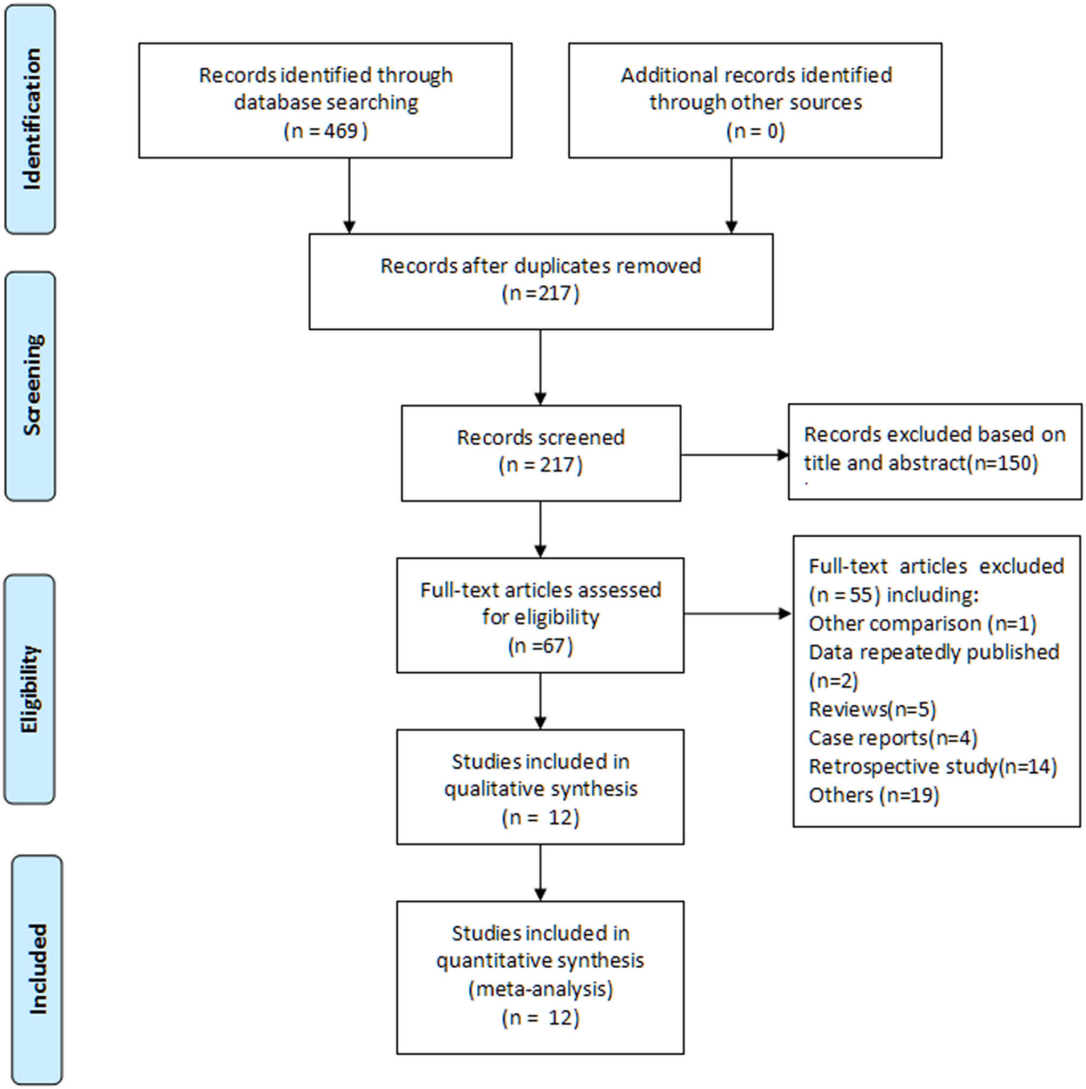
Flowchart of the literature screening and selection process.

A total of 469 articles were screened after a combined search of Embase, PubMed, Cochrane Library, CNKI, and Wanfang for articles published until February 1, 2020. Subsequently, 217 potential articles were selected after removing the duplicates. Of these, 150 articles were excluded based on title and abstract review, and the full-text of the remaining 67 articles was reviewed. An additional 55 articles were excluded according to the inclusion and exclusion criteria. Finally, 12 studies (1,013 patients; psychological nursing group: 509 patients; usual care group: 504 patients), published from 2004 to 2019, were included in this meta-analysis (16–27). The study selection process is illustrated in Figure 1.

### Characteristics of the Studies

**Figure 2:**
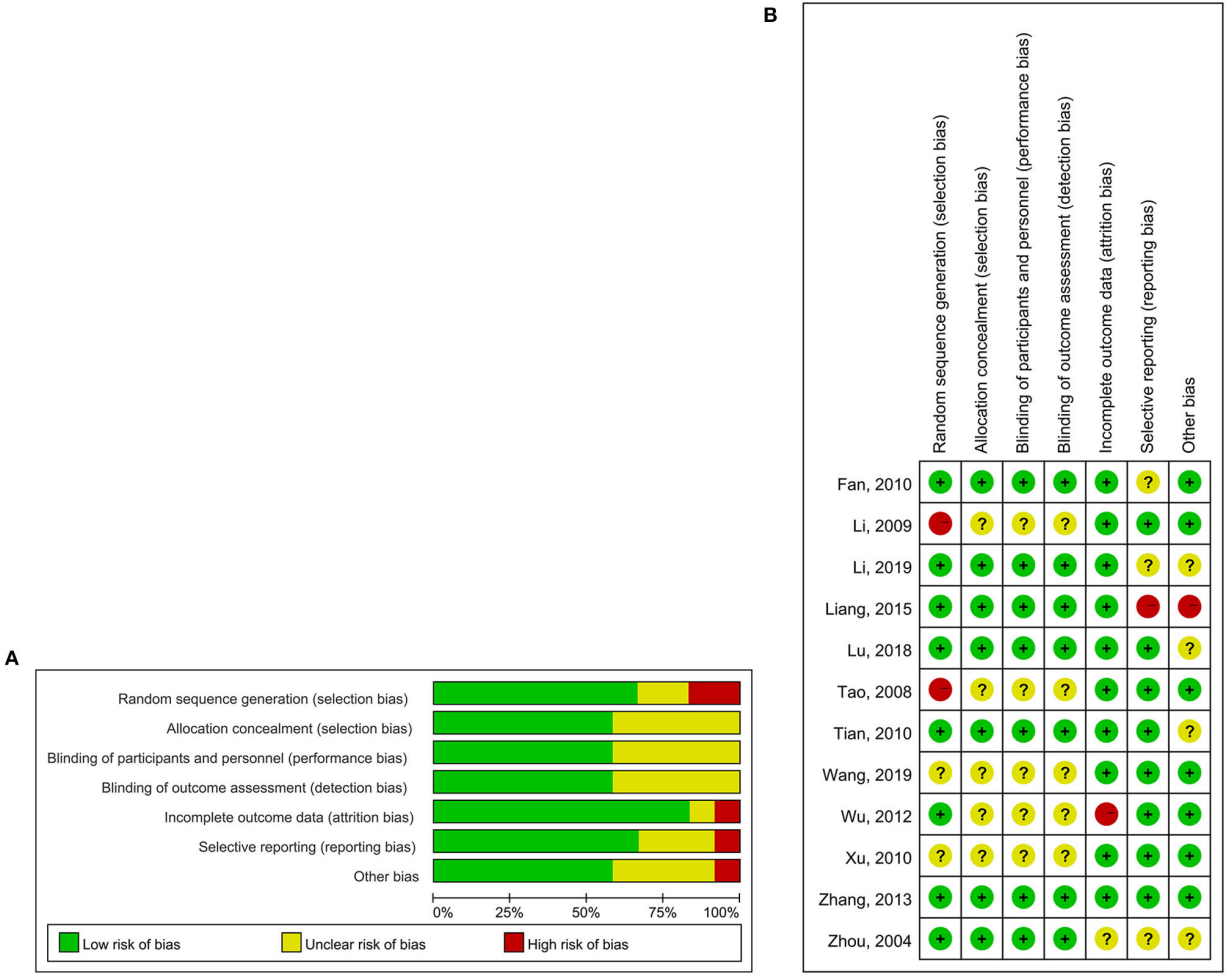
Quality assessment of included studies. **(A)** Risk of bias graph, **(B)** Risk of bias summary.

The sample size of the included studies was 50–114 cases, and the duration of follow-up was 2–8 weeks. The baseline characteristics of the 12 studies are summarized in Table 1. Three studies used an adequate method of random sequence generation by randomized code table (17, 19, 26). The quality of the included studies is summarized in Figures 2A,B.

**Table 1:** General characteristics of the included studies.

**Study, year**	***N***	**Male (%)**	**Experimental intervention**	**Control intervention**	**Measurements of primary outcomes**	**Mean age (years)**		
						**PN group**	**UC group**	**Follow-up**
Fan, 2010	80	43 (53.8)	Psychological care: Antidepressant treatment, cognitive psychological care, supportive therapy, behavior therapy, rehabilitation	Conventional symptomatic treatment	HAMD score	58.2	61.6	6 weeks
Li, 2009	114	82 (71.9)	Patients in treatment group were taken Psychological care + Conventional treatment Psychological care: 1. Establish a good relationship between nurses and patients 2. Supportive psychological care 3. Encouraging psychological nursing to eliminate patients' fear of disability 4. Music and reading therapy	Neurology conventional treatment and care	HAMD score	58	59.3	6 weeks
Li, 2019	60	29 (48.3)	Psychological care+Conventional treatment Psychological care: 1. Establish a good relationship between nurses and patients 2.Remove the patient's concerns.	Conventional treatment	HAMD score	63	64	NA
Liang, 2015	89	49 (55)	Psychological care+Conventional treatment Psychological care: 1. Remove the patient's concerns. 2. Reduce the patient's negative psychology and emotion and its impact on the prognosis of the disease 3. Provide patients with a sense of security and protect their self-confidence	Conventional treatment including reasonable diet, exercise and lifestyle, and correct bad life Habit and so on.	HAMD score	61.23	57.87	NA
Lu, 2018	60	37 (61.7)	Psychological care+Conventional treatment Psychological care: 1. Reduce the patient's negative psychology and emotion and its impact on the prognosis of the disease 2. Provide convenient, comfortable, and humanized services to create a comfortable, warm, and humanized ward 3. Provide patients with a sense of security and protect their self-confidence	Conventional antidepressant treatment and care	HAMD score	60.4	60.27	NA
Tao, 2008	62	36 (58)	Psychological care: 1. Reduce the patient's negative psychology and emotion 2. Eliminate patients' fear of disability	Conventional treatment	HAMD score	NA	NA	8 weeks
Tian, 2010	100	69 (69)	Psychotherapy care: 1. Behavioral intervention 2. Relaxation training and biofeedback therapy 3. Supportive psychotherapy 4. Strengthen communication and exchanges with family members of patients	Conventional treatment including decrease intracranial pressure, control blood pressure, general disease education, and so on.	HAMD score	NA	NA	3 weeks
Wang, 2019	50	31 (62)	Psychological care shown below: 1. Establish a good relationship between nurses and patients 2. Eliminate patients' fear 3.Humanistic education model	Conventional treatment	HAMD score	71.7	64.5	2 weeks
Xu, 2010	83	NA	Psychological care+ Conventional treatment Psychotherapy care: 1.Strengthen communication 2. Learn about diseases 3. Correct bad lifestyle	Conventional treatment	HAMD score	56.1	55	NA
Wu, 2012	120	62 (51.7)	Psychological intervention + Comprehensive rehabilitation training Psychotherapy care: 1. Early psychological intervention was given by professional psychologists 2. Early rehabilitation training including the paralysis limbs were well placed. Sitting position balance training to 3rd level; Standup and sitdown training; Standing position balance and movement training; Walking training.	Conventional treatment	SCL-90 score	56.1	56.7	3 weeks
Zhang, 2013	95	57 (60)	Psychological care+ Conventional treatment Psychotherapy care: 1. Supportive psychological care 2.Behavior therapy 3.Cognitive care	Conventional treatment	HAMD score	56.5	56.4	NA
Zhou, 2004	100	58 (58)	Psychological intervention + Comprehensive rehabilitation training Psychological intervention: 1. Provide patients with a sense of security and protect their self-confidence 2. Eliminate patients' fear of disability 3. Fully understand the role of rehabilitation	Conventional treatment: 1. Maintenance of good limb position 2. Sitting, standing, and walking training 3. Cognitive training 4. Acupuncture treatment 5. Music therapy 6. Strengthen nursing education	HAMD score	65.3	67.9	6 weeks

### Psychological Nursing of Patients With Stroke

**Figure 3:**
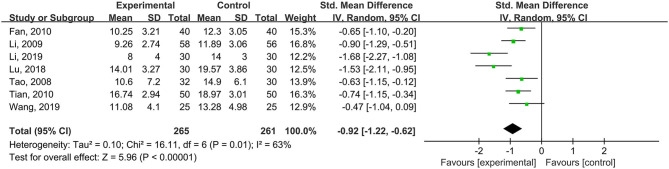
Meta-analysis of depressive symptoms assessed by the HAMD score.

**Figure 4:**
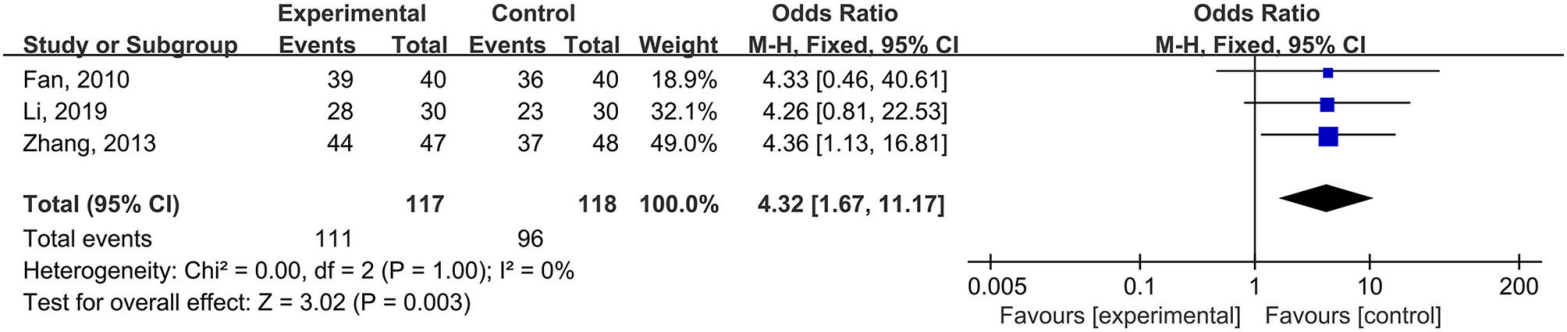
Meta-analysis of depressive symptoms assessed by the author self-defined.

Seven studies (*n* = 526 patients) used the total HAMD scale as the outcome measurement, and the results of the meta-analysis showed a significant difference in the HAMD score between the psychological nursing and the usual care groups (standardized mean difference (SMD) = −0.92, 95% CI: −1.22 to −0.62, *I*^2^ = 63%, *P*_heterogeneity_ = 0.01) (Figure 3). Meta-regression analyses revealed that the publication year (*P* = 0.642), sample size (*P* = 0.633), and mean age (*P* = 0.175) of the intervention group did not have moderating effects. The meta-analysis of three studies (*n* = 235) that used a depressive symptom control of ≥25% as the outcome revealed a significant difference between the two groups (OR = 4.32, 95% CI: 1.67–11.17, *I*^2^ = 0%, *P*_heterogeneity_ > 0.999) (Figure 4).

### Efficacy for Improving Neurological Rehabilitation by the NIHSS Scale

**Figure 5:**
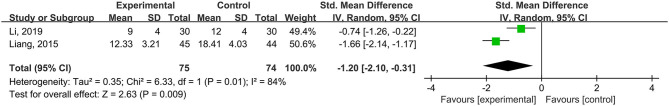
Meta-analysis of neurological rehabilitation by the NIHSS scale.

Two studies (*n* = 149 patients) used the NIHSS scale as the outcome. The results of the meta-analysis showed a significant difference in the NIHSS score between the psychological nursing and the usual care groups (SMD = −1.20, 95% CI: −2.10 to −0.31, *I*^2^ = 84%, *P*_heterogeneity_ = 0.01) (Figure 5). The meta-regression analyses revealed that the publication year (*P* = 0.46), sample size (*P* = 0.24), and mean age of the intervention group (*P* = 0.68) did not have moderating effects on the efficacy for improving neurological rehabilitation by the NIHSS scale.

### Efficacy for Recovering ADL

**Figure 6:**
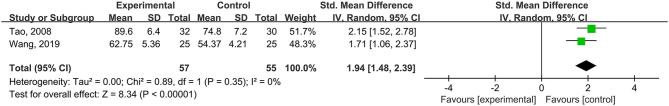
Meta-analysis of the ability of daily life by the ADL scale.

Two studies (*n* = 817 patients) used the ADL scale as the outcome. The meta-analysis showed a significant difference in the ADL score between the psychological nursing and the usual care groups (SMD = 1.94, 95% CI (1.48–2.39), *I*^2^ = 0%, *P*_heterogeneity_ = 0.35) (Figure 6).

### Publication Bias

**Figure 7:**
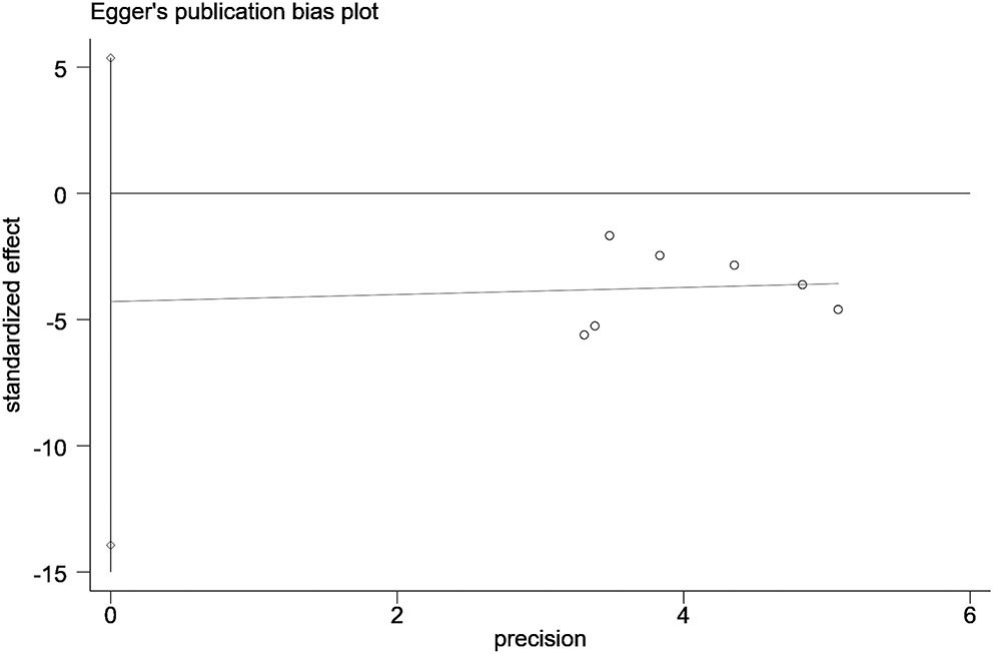
Funnel plot of meta-analysis.

Funnel plots and Egger's test did not show any publication bias across studies with respect to data on alleviating depressive symptoms, as assessed by the HAMD scale (*t* = −1.14, 95% CI: −13.9 to 5.36, *P* = 0.305) (Figure 7).

## Discussion

This systematic review and meta-analysis identified 12 studies investigating the psychological nursing of patients with stroke in China. The analysis found that compared to the usual care, psychological nursing is more effective for alleviating depressive symptoms, improving neurological rehabilitation, and recovering the ADL.

In the present meta-analysis, the HAMD, NIHSS, and ADL scores favored the psychological nursing group as compared to the usual care group. Nevertheless, all three scores examine different aspects. HAMD is a well-known and widely used questionnaire for depression and recovery (31). It examines mood, guilt, suicide ideation, insomnia, agitation or retardation, anxiety, weight loss, and somatic symptoms. NIHSS is a tool that allows the objective quantification of the impairments caused by stroke; also, it is applied to determine the severity of a stroke and examine recovery (32). ADL is used to determine the extent to which a patient is able to care for him/herself and determine the extent of the disabilities after stroke (33). Despite that these scales do not measure the same outcomes, all three are inter-related in patients with stroke (34–36). Patients with more severe stroke are more likely to have impairments in daily life activities and to have depression, patients with higher impairments are more likely to be depressed and present a lower score of NIHSS, and depressed patients putatively score low in the NIHSS and have low willingness to perform daily activities.

Several recent RCTs assessed the effects of psychological nursing of patients with stroke in China. These trials assessed the effects of psychological nursing on depressive symptoms, neurological rehabilitation, and the ADL. Li et al. (19) concluded that the HAMD and NIHSS scores of the psychological nursing group were significantly lower than those of the usual care group, and the total effective rate of the nursing intervention was 93% (28/30), which was significantly higher than the 77% (23/30) of the usual care group. Tian et al. (22) demonstrated that the HAMA and HAMD scores in the psychological nursing group were significantly lower than those in the usual care group after 3 weeks of intervention. Wu et al. (25) showed that psychological nursing significantly improved the mental health, limb movement function, stress ability, and the activity of daily living in patients with acute stroke. In the current meta-analysis, we observed a significant difference in the HAMD score, NIHSS score, and ADL scale between the psychological nursing and the usual care groups. The results demonstrated that psychological nursing is more effective than the usual care for alleviating depressive symptoms, improving neurological rehabilitation, and recovering ADL.

Those effects of psychological nursing on scales of anxiety, depression, and stroke-related impairments are consistent with the core aim of psychological nursing, i.e., improving the quality of life of the patients by decreasing anxiety and depression through a continuous and ongoing psychological care process, which is opposite to classical psychological interventions such as cognitive-behavioral therapy, which requires the participation of the patients and specialized healthcare professionals (11, 12). Decreased anxiety and depression are conducive to improved quality of life, which in turn is conducive to better functional outcomes and decreased health-related anxiety (34–36). Psychological nursing involves all the minute details in the nurse-patient relationship and that are related to the biological-psychological-social model paradigm of modern nursing care (13).

Nevertheless, the current study has some limitations. First, because of the differences between the healthcare systems in China and Western countries, a decision was made to include studies conducted only in China. Although this limits the generalizability, it improves the applicability of the results nationwide. Second, although all included studies were RCTs, the main limitation was the significant risk of reporting bias and selection, as well as the risk of bias evaluation. Third, the gray literature was not included. Fourth, the efficacy in long-term follow-up lacked evidence. Fifth, the majority of the included studies were from a single site using a small sample size, and hence, larger sample size and multicenter studies should be conducted. Finally, high heterogeneity was detected across some outcomes. This could be attributed to the articles with meta-analysis that investigated psychological nursing after stroke; however, the exact content of this psychological nursing varied across centers.

In conclusion, this systematic review and meta-analysis demonstrated that psychological nursing might be more effective for alleviating depressive symptoms, improving neurological rehabilitation, and recovering the ADL than the usual care group in China. Thus, well-designed and larger studies are essential to characterize the efficacy of psychological nursing for patients with stroke. Nevertheless, the present meta-analysis suggested that psychological nursing improves the psychological outcomes of patients after stroke and that such programs should be implemented in centers caring for patients with stroke. This should be conducive to reduce the burden on the healthcare system.

## Data Availability Statement

The original contributions presented in the study are included in the article/Supplementary Materials, further inquiries can be directed to the corresponding authors.

## Author Contributions

BL and ML conceived and supervised the study. QO and XC analyzed the data. BL, ML, and HS wrote the manuscript. YS and QO made manuscript revisions. All authors reviewed the results and approved the final version of the manuscript.

## Conflict of Interest

The authors declare that the research was conducted in the absence of any commercial or financial relationships that could be construed as a potential conflict of interest.
